# Synthesis and Characterization of Nanofibrous Polyaniline Thin Film Prepared by Novel Atmospheric Pressure Plasma Polymerization Technique

**DOI:** 10.3390/ma9010039

**Published:** 2016-01-11

**Authors:** Choon-Sang Park, Dong Ha Kim, Bhum Jae Shin, Heung-Sik Tae

**Affiliations:** 1 School of Electronics Engineering, College of IT Engineering, Kyungpook National University, Daegu 702-701, Korea; purplepcs@ee.knu.ac.kr (C.-S.P.); ao9o9@ee.knu.ac.kr (D.H.K.); 2 Department of Electronics Engineering, Sejong University, Seoul 143-747, Korea; hahusbj@sejong.ac.kr

**Keywords:** polyaniline, atmospheric pressure plasma, nanofiber, nanoparticle, plasma-polymerized aniline (pPANI), transmission electron microscopy (TEM), polycrystalline, intense plasma cloud type atmospheric pressure plasma jets (iPC-APPJ)

## Abstract

This work presents a study on the preparation of plasma-polymerized aniline (pPANI) nanofibers and nanoparticles by an intense plasma cloud type atmospheric pressure plasma jets (iPC-APPJ) device with a single bundle of three glass tubes. The nano size polymer was obtained at a sinusoidal wave with a peak value of 8 kV and a frequency of 26 kHz under ambient air. Discharge currents, photo-sensor amplifier, and optical emission spectrometer (OES) techniques were used to analyze the plasma produced from the iPC-APPJ device. Field emission scanning electron microscopy (FE-SEM), transmission electron microscopy (TEM), Fourier transform infrared spectroscopy (FT-IR), gas chromatography-mass spectrometry (GC-MS), and gel permeation chromatography (GPC) techniques were used to analyze the pPANI. FE-SEM and TEM results show that pPANI has nanofibers, nanoparticles morphology, and polycrystalline characteristics. The FT-IR and GC-MS analysis show the characteristic polyaniline peaks with evidence that some quinone and benzene rings are broken by the discharge energy. GPC results show that pPANI has high molecular weight (*M_w_*), about 533 kDa with 1.9 polydispersity index (PDI). This study contributes to a better understanding on the novel growth process and synthesis of uniform polyaniline nanofibers and nanoparticles with high molecular weights using the simple atmospheric pressure plasma polymerization technique.

## 1. Introduction

Recently, various nanomaterials, such as nanofibers, nanoparticles, nanowires, nanotubes, nanoribbons, *etc.*, have attracted a great deal of attention due to their technological advances in broadening the scope of applications, for instance, optoelectronics, molecular electronics, and bionanotechnologies [1,2,3]. The creation of new nanomaterials would require novel technological developments. Among various nanomaterials, polymer nanostructures have aroused great deal of interest because of the ease of the methods and their many advantages [4]. Polymerized nanoparticles and nanofibers can be prepared using various techniques, such as chemical synthesis, electrochemical method, electrospinning, ultrasonic irradiation, hard and soft templates, seeding polymerization, interfacial polymerization, and plasma polymerization [5,6,7,8,9,10,11,12,13,14,15,16,17]. Among these methods, plasma polymerization and aerosol-through-plasma (A-T-P) systems have versatile advantages, especially having “dry” process, for the deposition of plasma polymer films [3,18,19,20,21] and carbon based materials [22,23,24,25,26,27,28,29,30,31,32,33] with functional properties suitable for a wide range of applications, such as electronic and optical devices, protective coatings, and biomedical materials. Furthermore, it is well-known that plasma polymers are highly cross-linked, pinhole-free, branched, insoluble, and adhere well to most substrates.

Plasma polymerization is mostly performed at low pressure and radio frequency (RF) plasmas [34,35,36], limiting its application to batch processes. In addition, in low pressure plasma techniques, vacuum pumping systems are costly and require routine maintenance. Moreover, the allowed substrate size is limited by the chamber size. This has motivated the research and development of atmospheric pressure plasma techniques to simplify the experimental operations and to reduce apparatus cost and process time. Recently, atmospheric pressure plasma jets (APPJs) have been studied, mainly for the surface modification and biomedical application due to their low temperatures [37,38,39]. However, conventional APPJs have difficulty in realizing plasma polymerization due to the low plasma energy in ambient air conditions; as a result, they would not feed enough energy for activation to the monomers. In spite of intensive studies, only a few groups have reported successful plasma polymerization using APPJs [40,41,42,43,44]. Nonetheless, most plasma polymer films grown using the APPJs tend to show poor film qualities, such as low molecular weight and weak chemical stabilities, which would inherently result from the use of plasma with low density and electron temperature in monomer fragmentation (or active) and recombination (or passive) regions. In order to synthesize the polymer films using the APPJs, it is very important to increase the density and electron temperature of the plasma during plasma polymerization [40,41,42,43,44]. Therefore, this study aims at designing a new generation of plasma-polymerized aniline (pPANI) nanoparticles and nanofibers using a simple and new atmospheric pressure plasma polymerization technique with high plasma energy and ultrafast deposition process. Instead of measuring the plasma density and electron temperature, the photo-sensor amplifier and optical emission spectrometer (OES) techniques were used to analyze the optical intensity and spectrums of reactive nitrogen peaks, respectively, for estimating the variations in the plasma energy states [44,45,46,47,48]. 

In this study, we propose the intense plasma cloud type atmospheric pressure plasma jets (iPC-APPJ) device with a single bundle of three glass tubes to enhance the plasma jets in fragmentation or recombination regions during plasma polymerization process. In order to produce intense plasma cloud with high plasma density, we use a plastic tube and substrate on a polytetrafluoroethylene (PTFE) bottom cap, installed at the jet end, to minimize the quenching from ambient air and increase the plasma energy in the recombination region. Meanwhile, when using a single tube with a bottom cap or single bundle of three glass tubes without bottom cap, intense plasma clouds cannot be produced. Consequently, the iPC-APPJ including a bottom cap can extend farther downstream and can produce the broad and intense glow plasma in the fragmentation and recombination regions. 

Furthermore, the proposed iPC-APPJ can obtain a high molecular weight (MW) and high quality pPANI nanoparticles and nanofibers, with easily controllable morphology, deposition thickness, and size. Field emission-scanning electron microscopy (FE-SEM), transmission electron microscopy (TEM), Fourier transform infrared spectroscopy (FT-IR), gas chromatography-mass spectrometry (GC-MS), and gel permeation chromatography (GPC) techniques were used to analyze the pPANI.

## 2. Results and Discussion

### 2.1. Detailed iPC-APPJ Device and Optical, Electronical, and Discharge Characteristics

**Figure 1:**
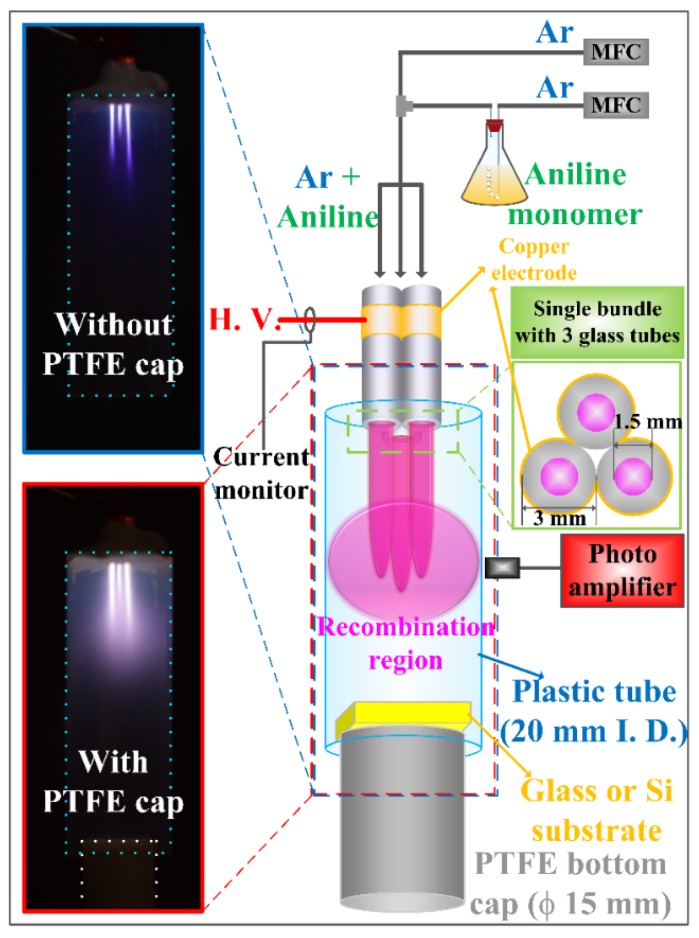
Schematic diagram of experimental setup in this study and images of plasmas produced in the recombination region of intense plasma cloud type atmospheric pressure plasma jets (iPC-APPJ), with and without the polytetrafluoroethylene (PTFE) bottom cap.

Figure 1 shows a schematic diagram of the proposed iPC-APPJ with the optical and electrical measurement system employed in this study. The three jet tubes, arranged in a triangle, were combined and wrapped through the powered electrode with copper tape, 10 mm from the end of the jet, which resulted in a more compact design, with each jet in physical contact with the adjacent jets. Each jet tube was 13 cm in length, with an inner diameter (i.d.) of 1.5 mm and an outer diameter (o.d.) of 3 mm, with the center-to-center distance between the two adjacent tubes at 3 mm. It is noted that the proposed plastic tube and PTFE bottom cap were installed at the jet end to confine the jet flow in the recombination region and to produce an intense plasma cloud. Moreover, it is well-advised to leave a gap between the end of the plastic tube and the substrate with the PTFE bottom cap for a smoother jet flow. A sinusoidal power supply was connected to the powered electrode with a peak value of 8 kV and a frequency of 26 kHz. As shown in the plasma jet images, with and without the PTFE bottom cap (Figure 1), in the case of a jet without the PTFE bottom cap, the short plasma plumes were only produced in the recombination regions, however, in the case of a jet with the PTFE bottom cap, strong plasma plumes were produced with an additionally broad glow plasma region in a farther downstream region. It can be inferred that the proposed PTFE bottom cap plays a role in minimizing the quenching from ambient air and in confining the jet flow in the recombination region by reducing exhaust gas flow.

**Figure 2:**
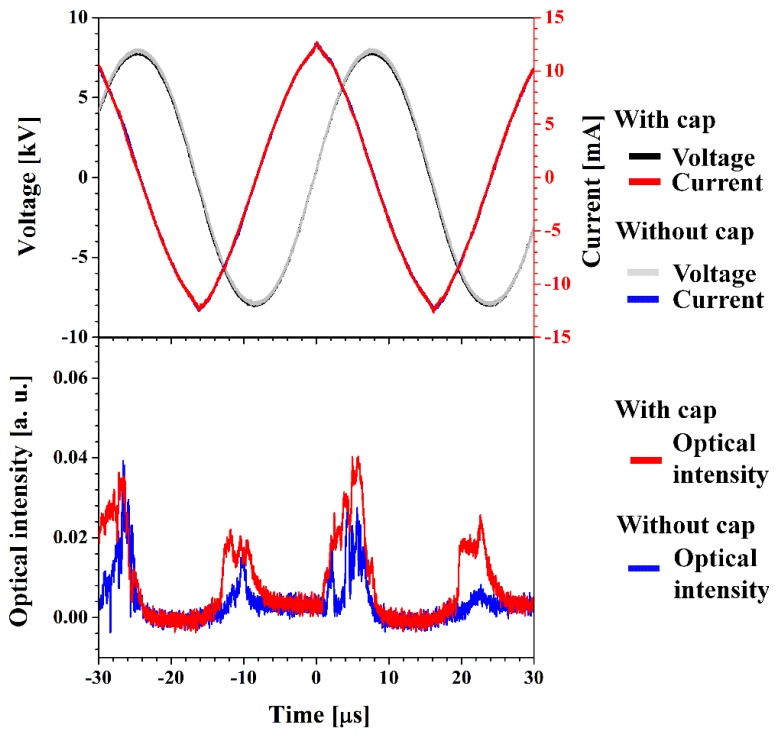
Applied voltages, discharge currents, and optical intensities measured in the recombination region of iPC-APPJ, with and without the PTFE bottom cap.

Figure 2 shows the applied voltages, discharge currents, and optical intensities measured in recombination region for proposed iPC-APPJ with and without PTFE bottom cap. As shown in Figure 2, the optical intensity was observed to have increased significantly due to the presence of the PTFE bottom cap without any changes of discharge voltage and current. It should be noted that there was a definite shift in the optical emission peak to the left, compared to that of the case without the PTFE bottom cap. It indicates that the proposed iPC-APPJ system can produce an efficient and intensive plasma jet due to the effect of the PTFE bottom cap, by analyzing optical intensities using a photo-sensor amplifier. Accordingly, the iPC-APPJ can produce a broad and intense glow plasma in the recombination regions during plasma polymerization.

**Figure 3:**
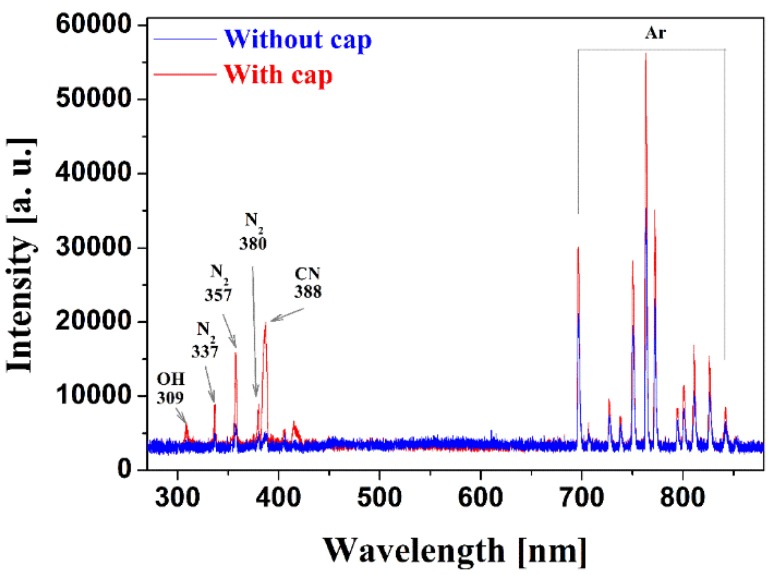
Optical emission spectra measured in the recombination region of iPC-APPJ, with and without the PTFE bottom cap.

Figure 3 shows the emission spectra from 270 to 880 nm, measured in the recombination region for the proposed iPC-APPJ with and without the PTFE bottom cap, further indicating that the excited N_2_, Ar, OH, and carbonaceous species exist in the plasma plumes. In the full spectrum measurements, significant increases in the various nitrogen (N_2_; 337, 357, and 380 nm) peaks and the oxygen (OH; 309 nm) peak were observed in the case with the PTFE bottom cap, while the Ar peak was slightly changed. It also indicates that the proposed iPC-APPJ system can produce an efficient and intensive plasma jet due to the effect of the PTFE bottom cap, by analyzing spectrums of various reactive species peaks using the OES technique [44,45,46,47,48]. The various N_2_ and OH peaks indicate a higher concentration of reactive nitrogen species (RNS) and reactive oxygen species (ROS), respectively, present in the iPC-APPJ with the PTFE bottom cap; both of these free radicals have been shown to play an important role in many biological/medical and industrial applications, and imply the presence of a more useful, efficient, and energy-dense plasma. In addition, spectra from carbonaceous species, such as CN (388 nm *B^2^Σ → X^2^Σ*), emitted during fragmentation and recombination processes of aniline monomer were only monitored for the case with the PTFE bottom cap. This means that the proposed iPC-APPJ can be suitable for sufficient fragmentation of the aniline monomer and an efficient generation of a new polymer in the recombination regions.

### 2.2. Functionalization and Characterization of Newly Synthesized pPANI Nanoparticles and Nanofibers Using iPC-APPJ

**Figure 4:**
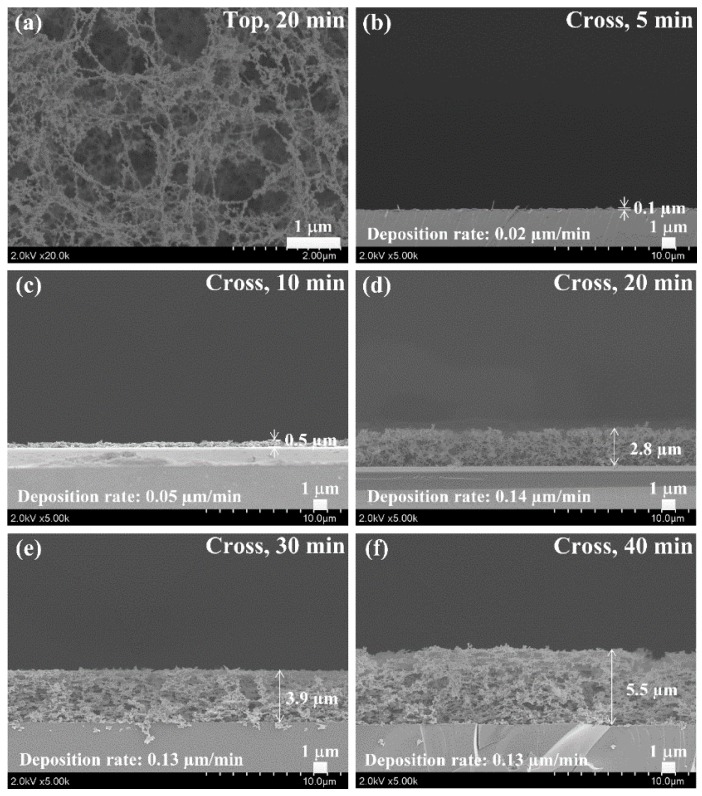
Scanning electron microscopy (SEM) images of plasma-polymerized aniline (pPANI) nanofibers with nanoparticles (NwN) thin film prepared via iPC-APPJ during various deposition times. (**a**) Top-view of pPANI after 20 min deposition; (**b**)–(**f**) cross-section view of pPANI at various deposition times. Scale bar = 1 µm.

Figure 4 shows the top-view and cross-section-view SEM images of the pPANI nanofibers with nanoparticle (NwN) thin film, grown for 5–40 min, on glass prepared using iPC-APPJ. No NwN were observed in the case without the PTFE bottom cap (not shown here). Whereas, in the case with the PTFE bottom cap, many NwNs were observed to be linked together in many irregular cross-linked networks, and porous networks were clearly observed in Figure 4a, meaning that the nanofibers and nanoparticles can be efficiently synthesized with the proposed iPC-APPJ. In addition, as shown in Figure 4a, the presence of pPANI nanofibers with a diameter range of 10–20 nm were clearly observed. As shown in Figure 4b–f, under various deposition times, the deposition rate was low during 5-min deposition. However, the deposition rates were increased significantly during 10-min deposition and maintained after 20 min at about 0.13–0.14 µm·min^−1^ in the case of the PTFE bottom cap, meaning that the nano-size polymer could be grown rapidly during the plasma polymerization with the proposed iPC-APPJ.

**Figure 5:**
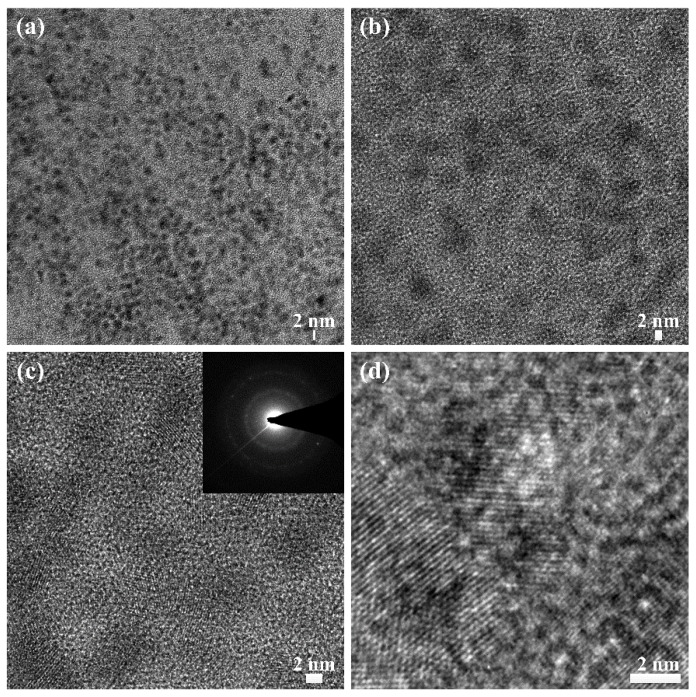
Transmission electron microscopy (TEM) images of pPANI nanoparticles thin film prepared via iPC-APPJ. (**a**–**d**) The different magnifications; the inset in (c) is the selected area electron diffraction (SAED) pattern of pPANI nanoparticles. Scale bar = 2 nm.

Figure 5 shows the TEM images of the pPANI nanoparticles prepared using iPC-APPJ. As shown in Figure 5, the pPANI nanoparticles, with a diameter range of 8–10 nm, were clearly observed. The particle sizes monitored using TEM are observed to be smaller than those measured using SEM, as expected for the dehydrated state of the TEM samples. The selected area’s electron diffraction (SAED) pattern of pPANI nanoparticles (Figure 5c, inset) reveals the clear diffraction ring and spot of the polycrystalline characteristics [49,50,51,52]. The polycrystalline characteristics of pPANI nanoparticles may be caused by many irregular cross-linked networks, characteristic of plasma polymerization. The TEM analysis of Figure 5 confirms that the plasma polymerization using the proposed iPC-APPJ can produce uniform pPANI nanoparticles of a very small size and polycrystalline characteristics. 

**Figure 6:**
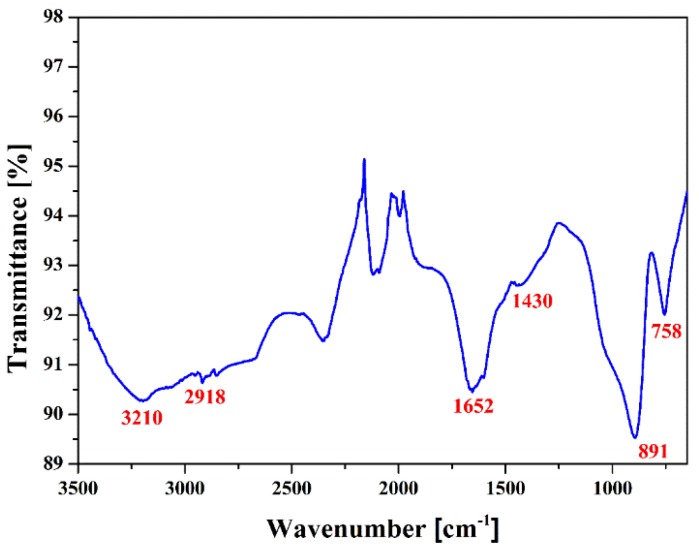
Fourier transform infrared spectroscopy (FTIR) spectrum of pPANI NwN thin film prepared using iPC-APPJ.

Figure 6 shows the Fourier transform infrared spectroscopy (FTIR) spectrum of the pPANI nanofibers and nanoparticles thin film synthesized using the iPC-APPJ technique. As shown in Figure 6, FTIR spectrum was performed using the transmittance method since the pPANI films grown in this experiment were too thin to use the absorbance method. The characteristic peaks of the emeraldine base form of pPANI were observed in the broad peak at 3210 cm^−1^ (N-H stretching with hydrogen bonded 2° amino groups) and 1430 cm^−1^ (C–C stretching vibration of benzenoid rings). A peak belonging to the C–H aliphatic vibration was located at 2918 cm^−1^. A peak at 1652 cm^−1^ was due to the quinonoid structure of pPANI. The out-of-plane C–H ring bending vibration was found in the sharp peak at 891 cm^−1^ and 758 cm^−1^. The films with better performance in electric conductivity were located in these peak regions [14]. These related peaks confirm the successful formation of PANI. This observation suggests that the produced pPANI films are more likely in the emeraldine base form.

**Figure 7:**
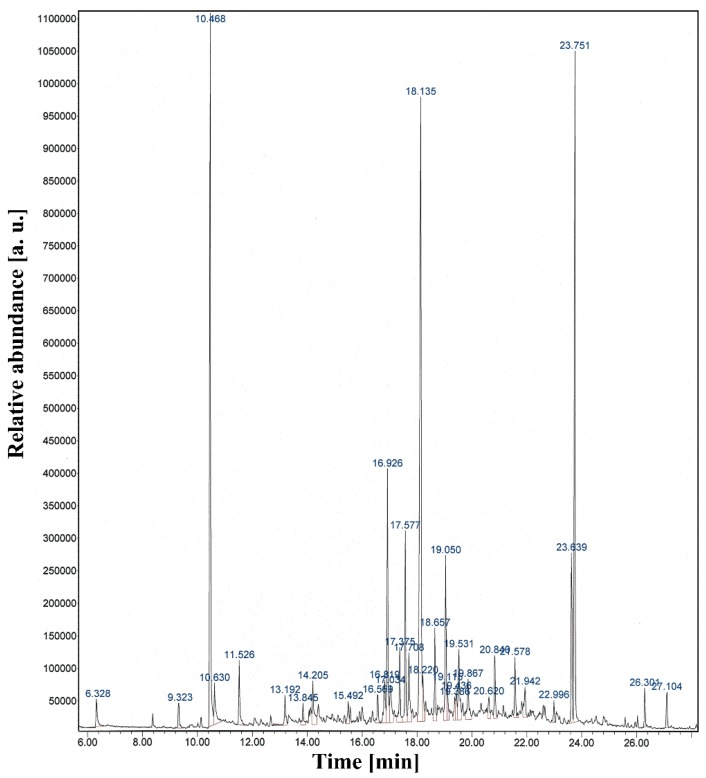
Total ion count chromatogram of pPANI NwN thin film obtained using solid-phase microextraction gas chromatography-mass spectrometry (SPME-GC-MS) prepared using iPC-APPJ.

Figure 7 shows the total ion count chromatogram of the pPANI NwN thin film obtained using solid-phase microextraction (SPME) GC-MS. As shown in Figure 7, the peaks at 10.468 min and 16.926 min were related to benzenamine and benzonitrile, respectively. The peak at 23.751 min was related to N=N bending vibration of azobenzene. FT-IR and GC-MS analysis show the characteristic polyaniline peaks, with evidence that some quinone and benzene rings were broken in the recombination region due to the intensive plasma jet. It also notes that our pPANI nanofibers and nanoparticles have an insulating state with the emeraldine base form, partially oxidized, with a few N–H groups in the main chain. However, emeraldine can easily change from insulator to conductor when it is protonated with a proton donor, such as HCl and iodine (I_2_) [13,14,34,35,53,54].

Table 1 summarizes the weight average MW (*M_w_*) of the aniline monomer and *M_w_*, number average MW (*M_n_*), and polydispersity index (PDI) of the pPANI NwN thin film synthesized using the iPC-APPJ technique. As shown in the GPC results of Table 1, the synthesized pPANI showed high molecular weights (*M_w_*) of about 533 kDa and an excellent PDI of about 1.9. Consequently, these experimental results confirm that the proposed iPC-APPJ device, driven by the sinusoidal voltage waveform, can obtain high molecular weight and ultra-fast (during just 10–30 min) pPANI films and NwN. Furthermore, we expect that our pPANI NwN can provide a unique advantage for optoelectronics, molecular electronics, and bionanotechnology applications. In addition, this proposed device is expected to be used for plasma polymerization with various monomers.

**Table 1:** Summary of calculated molecular weights of pPANI NwN thin film obtained by gel permeation chromatography (GPC) prepared using iPC-APPJ.

Samples	*M_w_* (kDa)	*M_n_* (kDa)	Polydispersity Index (PDI)
Aniline monomer (Aldrich)	0.093	-	-
pPANI	533.2	283.0	1.9

## 3. Experimental Section 

### 3.1. Plasma Polyaniline Synthesis

The high purity argon gas (99.999%) was used as the discharge gas for plasma generation and its flow rate was 1700 standard cubic centimeters per minute (sccm). Liquid aniline monomer (Sigma-Aldrich Co., St. Louis, MO, USA, *M_w_* = 93 g·mol^−1^) was vaporized by means of a glass bubbler, which was supplied by the argon gas, with a flow rate of 160 sccm. The nano-sized polymer was obtained at a sinusoidal wave, with a peak value of 8 kV and a frequency of 26 kHz using the newly proposed iPC-APPJ device under ambient air.

The substrates used for the plasma polymerization were Si wafers, polyethylene terephthalates (PETs), and glasses. Before plasma polymerization, the slide glass was ultrasonically cleaned in 99.99% acetone, isopropanol, and distilled (DI) water for 20 min, respectively, so as to remove contamination on the surface of the substrates.

### 3.2. Optical and Electronical Measurement

A high voltage probe (Tektronix P6015A) was connected between the power supply and the oscilloscope (LeCroy WaveRunner 64Xi) to measure the sinusoidal voltage. In the driving circuit, an inverter was used to amplify low primary voltage to a high secondary voltage. The driving circuit generated a sinusoidal voltage of several tens of kilovolts, with a frequency of several tens of kilohertz. The photo-sensor amplifier (Hamamatsu C6386-01) was used to measure the plasma infrared (IR) emission in the recombination region. The wavelength-unresolved optical emission waveform from the photo-sensor amplifier was plotted on an oscilloscope. In front of the photo-sensor amplifier, a 1-mm-thick glass sheet was placed, in order to avoid external light signals, and the resulting distance between the end of the device and the measurement lead was about 20 mm. All photographs of the devices and plasma plumes were taken with a DSLR camera (Nikon D56300) with a Macro 1:1 lens (Tamron SP AF 90mm F2.8 Di). An optical emission spectrometer (OES, Ocean Optics, USB-4000UV-VIS) was employed to identify the diverse reactive species in the plasma.

### 3.3. Field Emission Scanning Electron Microscopy

The morphology and cross-sectional images of plasma polyaniline nanofibers were characterized using field emission scanning electron microscopy (FE-SEM, Hitachi SU8220).

### 3.4. Transmission Electron Microscopy

The high-resolution transmission electron microscopy (HRTEM) images and selected area electron diffraction (SAED) patterns were taken with a Titan G2 ChemiSTEM Cs Probe (FEI Company, Hillsboro, OR, USA) transmission electron microscope, operating at 200 kV. A TEM sample was prepared by depositing a 6-µL solution of pPANI nanofibers with nanoparticles (NwN) (ultrasonically dispersed in DI water) on carbon-coated copper grids, and dried in air. 

### 3.5. Fourier Transform Infrared Spectroscopy

Fourier transform infrared spectroscopy (FT-IR) of these polymers were taken with a Perkin-Elmer Frontier spectrometer between 400 and 4000 cm^−1^, sampled directly from the films.

### 3.6. Gas Chromatography-Mass Spectrometry

An Agilent 7890A series GC, equipped with a split and splitless injector and a 5975C mass-selective detector system, was used to determine the optimized SPME conditions. In our experiment, poly(dimethylsiloxane) containing divinylbenzene (PDMS-DVB) was considered as a less polar coating. The MS was operated in the EI mode (70 eV). Helium (99.999%) was employed as carrier gas, and its flow rate was adjusted to 1 mL·min^−1^. The separation of pPANI was carried out using a capillary column HP-5 MS (30 m, 0.25 mm i.d.) with a film thickness of 0.25 µm. The gas chromatograph was operated in the split mode (split ratio 10:1). The GC column temperature was programmed at 40 °C for 3 min and then raised to 300 °C at 7 °C min^−1^, and kept at this temperature for 20 min. The injector temperature was set at 250 °C.

### 3.7. Gel Permeation Chromatography

Gel permeation chromatography (GPC) was performed on an Alliance e2695 (Waters, Milford, MA, USA) GPC system, equipped with a Styragel guard column, a Waters Styragel HR3 (molecular weight range: 5.0 × 10^2^–3.0 × 10^4^ g·mol^−1^), a Waters Styragel HR4 (molecular weight range: 5.0 × 10^3^–6.0 × 10^5^ g·mol^−1^), and a Waters Styragel HR5E (molecular weight range: 5.0 × 10^4^–4.0 × 10^6^ g·mol^−1^). Detection was performed on a 2414 refractometer using dimethylformamide (DMF) (high purity liquid chromatography (HPLC) grade, containing 1 mg·mL^−1^ LiBr) as the eluent at a flow rate of 1.0 mL·min^−1^. The temperature of the columns was set at 35 °C. Analysis of molecular weight and polydispersity index of polymers was performed using Empower 2 software against polystyrene standards (molecular weight range: 1.0 × 10^3^–2.6 × 10^6^ g·mol^−1^). Ultra-pure tetrahydrofuran (THF, 99.9% HPLC grade) was used as an eluent, as well as a solvent, to prepare the pPANI solutions. The polymer solutions was filtered through a 0.45 m PTFE syringe filter prior to use in order to prevent pressure fluctuation in the GPC system.

## 4. Conclusions

In summary, this study aims at designing a new generation of plasma-polymerized aniline (pPANI) nanofibers and nanoparticles using the simple atmospheric pressure plasma polymerization technique. The plasma-polymerized aniline (pPANI) nanofibers and nanoparticles are successfully obtained by the newly proposed intense plasma cloud type atmospheric pressure plasma jets (iPC-APPJ) device with a single bundle of three glass tubes. The experimental results show that the pPANI has high molecular weights and sizes of a few tens of nanometers. It also notes that our pPANI nanofibers and nanoparticles have an insulating state with an emeraldine base form. Emeraldine can easily change from insulator to conductor when it is protonated with a proton donor, such as HCl and iodine (I_2_). A detailed parametric study should be done to optimize molecular structure and increase electrical conductivity of the nano-size plasma polymer produced with this method in the future.
